# Association of Agriculture Occupational Exposure With Diabetes and Cardiovascular Risk Factors in South Indian Villages: REDSI Study

**DOI:** 10.3389/fcvm.2021.737505

**Published:** 2021-09-24

**Authors:** Ganesan Velmurugan, Sundaresan Mohanraj, Jenifer Christy Yacob, Sundaravadivu Keppanan, Balakrishnan Rekha, Anbalagan Krishnasamy, Suresh Shanmugarajan, Seenivasan Boopathi, Anitha Ayyapparaja, Prabhu Chandhran Ayyapparaja, Tamilselvan RS, Manigandan Gopalakrishnan, Jayaramanathan Veerappan, Vithya Dharmaraj, Subramaniyan Vaithilingam, Priyadharshini Purushothaman, Sumathi Chelladurai, Jeevan Pandiyan, Vijaya Samoondeeswari Selvarajan, Kalidoss Annathurai, Sukumaran Vengatachalam, Gorky Arivuruvone, Saravanan Kaliyaperumal, Velsamy G, Kannan S, Subbiah Ramasamy, Krishnan Swaminathan

**Affiliations:** ^1^Chemomicrobiomics Laboratory, Department of Biochemistry & Molecular Biology, KMCH Research Foundation, Coimbatore, India; ^2^Department of Molecular Biology, School of Biological Sciences, Madurai Kamaraj University, Madurai, India; ^3^Department of Environmental Sciences, School of Energy, Environment and Natural Resources, Madurai Kamaraj University, Madurai, India; ^4^Department of Medical Lab Technology, Dr. NGP Arts & Science College, Coimbatore, India; ^5^Department of Management Studies, Senthamarai College of Arts & Science, Madurai, India; ^6^Department of Microbiology & Biotechnology, JJ College of Arts & Science, Pudukottai, India; ^7^Department of Zoology, Kundavai Naacchiyaar Govt. Arts College for Women, Thanjavur, India; ^8^Department of Biotechnology, Marudhupandiyar College, Thanjavur, India; ^9^Department of Zoology, Nehru Memorial College, Tiruchirappalli, India; ^10^Department of Zoology, Aringar Anna College of Arts & Science, Krishnagiri, India

**Keywords:** diabetes, rural health, epidemiology, occupational exposure, agrochemicals

## Abstract

There has been a huge increase in diabetes and its associated cardiovascular complications over the last decade, predominantly in the middle- and low-income countries. In these countries, the majority live in rural areas. The Rural Epidemiology of Diabetes in South India (REDSI) study was aimed to analyze the prevalence of diabetes, cardiovascular risk factors, and its complications in rural farming and non-farming villages in Tamil Nadu, South India. A research survey on the prevalence of self-reported diabetes, cardiovascular risk factors (age, sex, obesity, hypertension, hypercholesterolemia, alcohol and tobacco use) and agricultural occupational exposure was executed among 106,111 people from 61 villages in the state of Tamil Nadu, South India, during 2015–2018. Overall, we observed a diabetes prevalence of 11.9% in rural South India. A nearly two-fold higher prevalence of diabetes was observed among the farming community (15.0%) compared to that among the non-farming population (8.7%). Logistic regression analyses revealed a strong association with agrochemical exposure (*P* < 0.0001) and diabetes prevalence among rural farming people. Our survey indicates a high prevalence of diabetes in rural South India particularly among the farming community. This survey in conjunction with other epidemiological and experimental studies raises the need for understanding the etiology of diabetes and other cardiovascular risk factors in rural communities.

## Summary

### What Is Already Known?

- As per a WHO report (2016), the increase in diabetes prevalence was particularly higher in middle- and low-income countries, which are dominated by rural communities (~70%).
- Rural people are thought to be relatively less susceptible to diabetes as they have fewer exposure to traditional risk factors for diabetes (physical inactivity, high fat diet, sugary/fizzy drinks, familial diabetic history, mental stress, etc.).
- Mounting evidence indicates the etiological role of endocrine-disrupting chemicals (EDCs) in diabetes prevalence. Agrochemicals, including pesticides and synthetic fertilizers, are the major source of EDCs in rural world.

### What This Study Adds?

- There is a high prevalence of diabetes among farming people (15.0%) in comparison to that among non-farming people (8.7%) in the study population of 106,111 people from rural South India.
- Among the traditional risk factors, only age and hypertension showed association with diabetes prevalence among the farming population while no association with other traditional factors (sex, obesity, hypercholesterolemia, alcohol intake, smoking, tobacco use) was observed.
- A statistically strong association of diabetes prevalence with occupational exposure to agrochemicals among the rural farming population was observed in this study.

## Introduction

The world has witnessed a massive increase in the prevalence of diabetes and its complications during the period 1980–2014 (1). The increase in diabetes rate during this period has not been similar in all nations (1, 2). The epidemic of diabetes prevalence is of several orders of magnitude especially in middle- and low-income countries, which are predominantly occupied by rural communities (2). It has been generally thought that urbanization, westernization, and affluence have significant roles in the explosion of diabetes, but we believe that these factors alone could not explain the diabetes epidemic in middle- and low-income countries, especially in rural areas. Intuitively, one would expect a low prevalence of diabetes in a rural population, where adherence to traditional lifestyles includes more physical activity and access to a more nutrient-rich (less processed foods) diet. In recent years, studies from different regions of the world indicated the increasing prevalence of diabetes and other metabolic diseases in rural communities (3–8).

India can be considered as an ideal reference for rural community, as 67% of its population still live in villages. In addition, India houses 17% of the world population and 15.3% of the global diabetes population (1, 2). India witnessed a large increase in diabetes population during 1980–2014 (4.6% increase), but during the same period, no similar significant increase in the level of common risk factors like obesity, hypertension, hypercholesterolemia, and smoking was observed in India, (2). Concurrently during the period of escalation of diabetes incidence, the world has witnessed the massive production and release of toxic chemicals into the environment (2). At present, there is a growing body of evidence that suggests an important role for endocrine-disrupting chemicals (EDCs) in the etiology of diabetes and cardiovascular disorders (2, 9, 10). The major source of EDCs in rural community is exposure to agrochemicals, which include different pesticides (organochlorines, organophosphates, carbamates, pyrethroids, neonicotinoids, etc.) and synthetic fertilizers, which are rich in toxic heavy metals like arsenic and cadmium (8). Epidemiological and experimental studies proved the role of agrochemicals in the development of insulin resistance, glucose intolerance, and pancreatic beta-cell disruption (2–10). The aim of this study is to assess the prevalence of self-reported diabetes and cardiovascular risk factors in a large rural community in South India and in parallel to unravel the association of diabetes and its complications with occupational exposure to agrochemicals.

## Methods

### Study Population

**Figure 1:**
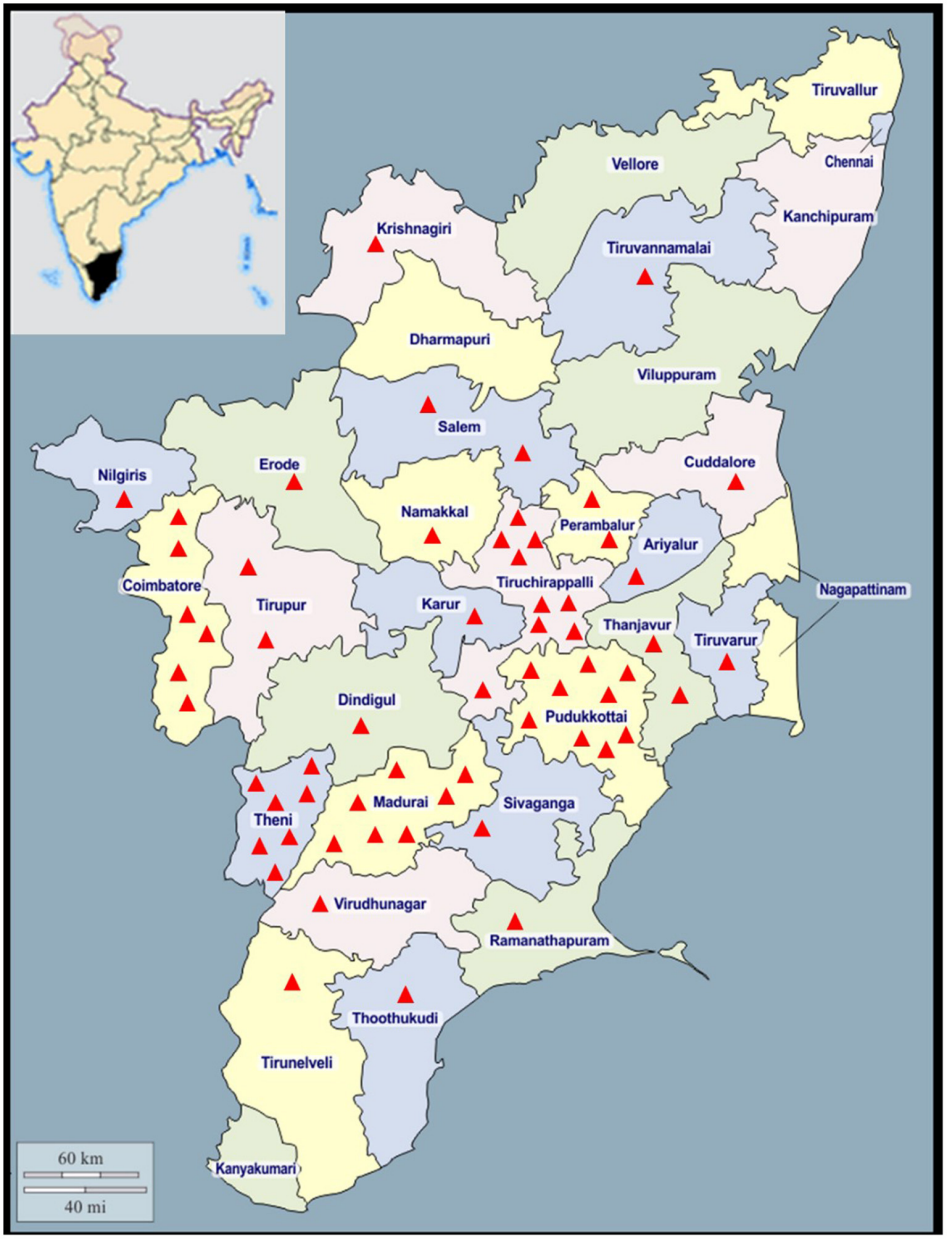
Geographical locations of the 61 study villages in the state of Tamil Nadu, India. Each red triangle represents a village. The sizes of the triangles are not to scale.

Sixty-one villages (comprising 106,111 people) representing 0.25% of the rural population in Tamil Nadu, India, was selected by a stratified process representing most of the major districts of Tamil Nadu (Figure 1) with populations ranging from 800 to 4,000 as per the recent census by the Government of India. Before starting the survey, the basic details of the village, including population, farming practices, sanitation, water resources, and health facilities, were collected from the village administrative office.

### Survey Design and Development

In consultation with our research team and medical practitioners, a pilot questionnaire was designed and this questionnaire was pilot tested in the first 13 villages of the study. The questionnaire structure was modified based on the inputs from the surveyors and the rural public. The final revised questionnaire was developed as an Android-based mobile application for the survey process. The mobile application was named as “Rural Diabetes Survey” and made available in Google Play Store but needed authorization from the administrator to enter data. The questionnaire includes age, sex, lifestyle risk factors (alcohol intake, smoking, and use of oral tobacco), metabolic risk factors (obesity, hypertension, and hypercholesterolemia), occupation, and exposure to agrochemicals (pesticide applicator, directly handles agrochemicals, field worker, no direct exposure). In addition, the complications of diabetes, including cardiovascular diseases, kidney failure, cancer, tuberculosis, diabetic retinopathy, and diabetic neuropathy, and history of other diseases were collected using a standard questionnaire provided in the mobile app. The study was named as “Rural Epidemiology of Diabetes in South India” (REDSI) study.

### Collection of Survey Data

Student volunteers were trained and dummy data were entered using the mobile app, and quality assurance was carried out. The survey process is a door-to-door strategy, and all the houses in the selected villages were surveyed. All the inhabitants of the selected villages aged above 18 years were included for the survey. The study protocol was approved by the institute ethical committee, and the purpose of the study was explained to the study population before the execution of survey.

### Validation of Survey Data

The self-reported diabetes and other diseases were validated in 12.8% (*n* = 13,649) of the population by direct review of their medical records and medications in a blinded fashion by our team who are not involved in the survey process. In case of self-reported obesity, the height and weight were measured directly using a calibrated weighing scale and measuring tapes. The body mass index (BMI) was calculated by standard protocols, and those with BMI ≥ 25 kg/m^2^ were categorized as generalized obesity. We are able to cross validate self-reported diabetes (by reference to fasting blood glucose, glycated hemoglobin, and/or medications), hypertension, hypercholesterolemia, cardiovascular diseases (myocardial infarction, stroke), kidney failure, cancer, and tuberculosis by reference to medical records and medications. The data on self-reported diabetic complications like diabetic retinopathy, diabetic neuropathy, and other frequently reported diseases like ulcer, asthma, kidney stones, hypothyroidism, and joint pains were not largely validated by reference to medical records and hence not included in analysis.

### Statistical Analysis

All statistical analyses were performed using the statistical software SPSS V.24.0. The study population was stratified on different criteria primarily based on occupational exposure to agrochemicals and categorized as farming and non-farming population. Baseline characteristics were determined separately for these strata, and differences were investigated using chi-squared test for categorical covariates. One-way ANOVA with Bonferroni *post-hoc* analysis was used to compare the prevalence of diabetes between different populations. Statistical significance was determined on the basis of two-sided *p*-values of < 0.05. Binary logistic regression was performed to study the association of self-reported disease status with different risk factors. Our logistic regression models were fitted with appropriate degrees of adjustment. Age, sex, alcohol, smoking and tobacco usage, obesity, hypertension, and hypercholesterolemia were used as confounder for adjustment in logistic regression. No data points were excluded from analyses. *P* < 0.05 was considered statistically significant in all analyses. ^*^P < 0.05, ^**^P < 0.01, ^***^P < 0.001, ^****^P < 0.0001. The bars and error bars represent mean and standard deviation, respectively.

## Results

A total of 106,111 adult individuals from 61 villages in Tamil Nadu (Figure 1) were surveyed in the REDSI study using a mobile app. The adult population of the villages ranged from 728 to 3,485, and the average population per village was 1,751 people. The characteristics of the study population are described in Table 1. We had equal representation of both sexes, and 32% of the population were below 35 years and 10% of the population were aged more than 65 years (Table 1). Of the total population, 51.7% are involved in farming activities, and they are sub-categorized into pesticide applicators, agrochemical handlers, and field workers based on their level of exposure to agrochemicals. In overall, 11.9% of self-reported diabetes prevalence was observed. Among the diabetes complications, we observed 1.9, 1, 0.3, and 0.2% of known CVDs, kidney failure, cancer, and tuberculosis in the rural population. Although there is no significant difference in the prevalence of diabetes complications between farming and non-farming communities, the increase in kidney failure was the highest among the farming community (Table 1).

**Table 1:** Characteristics of the study population categorized by occupational exposure to agrochemicals.

**Factors**		**Total population (%)**	**Occupation**		***P*-value**
			**Farming population (%)**	**Non-farming population (%)**	
Sex	Male	50.5	51.2	49.8	0.776
	Female	49.4	48.7	50.1	
	Transgender	0.1	0.1	0.1	
Age group	18–35	32.0	24.3	40.3	<0.05
	36–50	34.0	37.4	30.3	
	51–65	24.2	27.3	20.7	
	>65	9.8	10.9	8.7	
Occupation	Farming	51.7	100.0	-	-
	Non-farming	48.3	-	100.0	
Diabetes	Self-reported	11.9	15.0	8.7	<0.001
	Undiagnosed/no diabetes	88.1	85.0	91.3	
**Lifestyle risk factors**					
Alcohol intake	Daily	6.5	6.6	6.3	0.786
	Occasionally/formerly	11.4	13.2	9.7	
	Never	82.1	80.2	84.0	
Smoking	Daily	9.2	9.7	8.8	0.236
	Occasionally/formerly	4.6	5.5	3.7	
	Never	86.1	84.8	87.4	
Tobacco use	Daily	7.2	7.4	7.1	0.845
	Occasionally/formerly	4.5	5.7	3.3	
	Never	88.3	87	89.6	
**Metabolic risk factors**					
BMI	Obesity	10.7	10.7	10.7	0.521
	Normal	70.1	68.8	71.3	
	Underweight	19.2	20.5	18.0	
Blood pressure	Hypertension	12.0	14.6	9.5	<0.01
	Undiagnosed/normal	88.0	85.4	90.5	
Blood cholesterol	Hypercholesterolemia	2.5	3.3	1.8	<0.01
	Undiagnosed/normal	97.5	96.7	98.2	
**Agricultural occupational risk factors**					
Exposure to agrochemicals	Pesticide applicator	11.8	23.8	-	-
	Agrochemical handler	7.5	14.5	-	
	Field worker	30.7	61.7	-	
	No direct exposure	50.0	-	100.0	
**Diabetes complications**					
CVDs	Diagnosed CVDs	1.9	1.6	2.1	0.742
	Undiagnosed/no CVDs	98.1	98.4	97.9	
Kidney failure	Diagnosed kidney failure	1.0	1.6	1.4	0.521
	Undiagnosed/no	99.0	99.4	99.6	
Cancer	Diagnosed cancer	0.3	0.2	0.3	0.095
	Undiagnosed/no cancer	99.7	99.8	99.7	
Tuberculosis	Diagnosed TB	0.2	0.1	0.2	0.082
	Undiagnosed/no TB	99.8	99.1	99.8	

**Figure 2:**
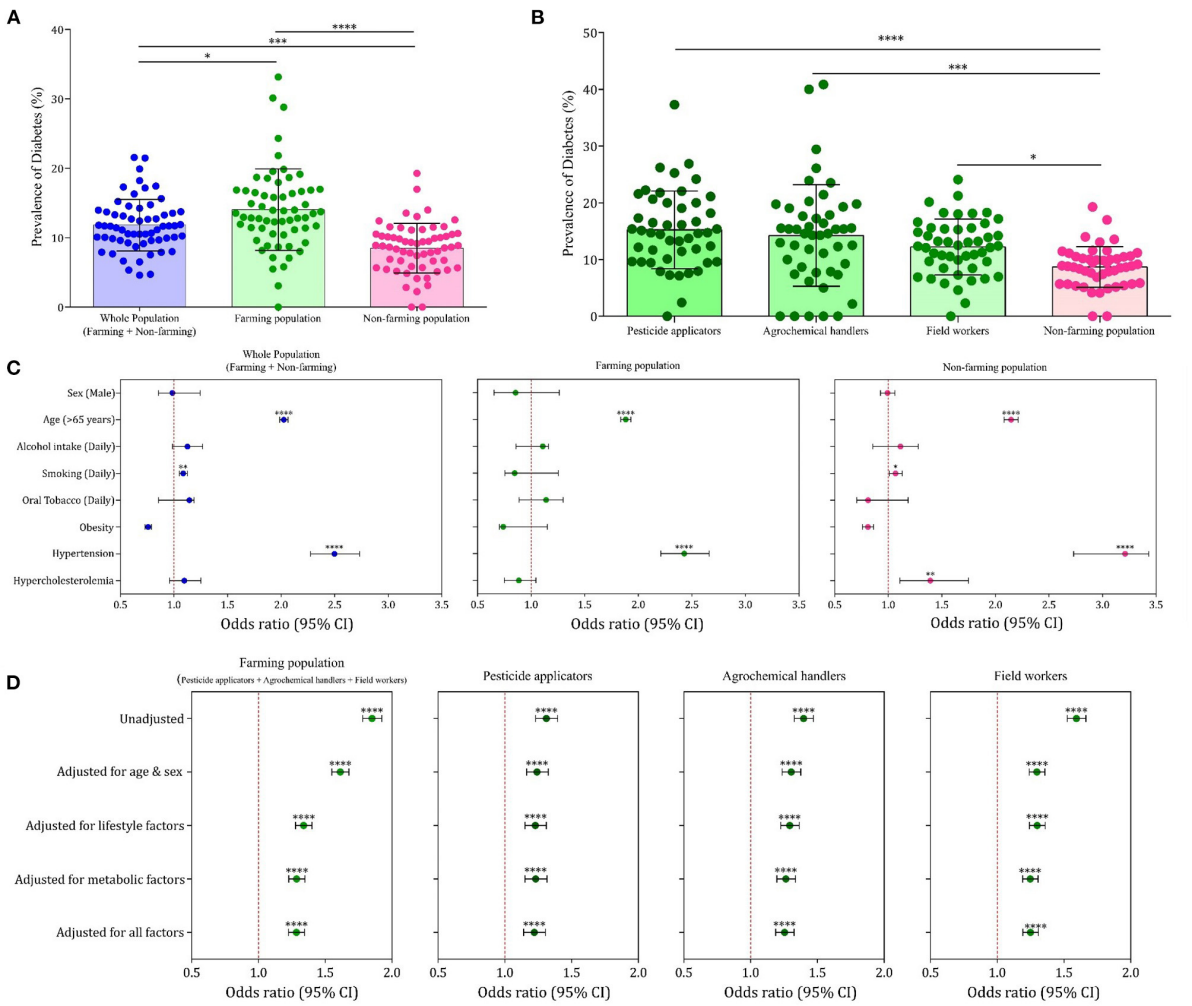
Rural epidemiology of diabetes in South India (REDSI) study. **(A)** Prevalence of diabetes among the whole, farming, and non-farming populations in the 61 villages studied. Each point represents the prevalence data from a single village. (**B)** Prevalence of diabetes among the people with categories of occupational exposure to agrochemicals. **(C)** Forest plot representing association of traditional risk factors with diabetes prevalence in total population and farming and non-farming populations. (**D)** Forest plot representing association of occupational exposure to agrochemicals and self-reported diabetes in farming people with different degrees of adjustment. *P < 0.05, **P < 0.01, ***P < 0.001, ****P < 0.0001.

On further analysis based on occupation, the prevalence of diabetes was 15.0% among the farming community (occupationally exposed to agrochemicals) and 8.7% among the non-farming community (*P* < 0.0001, Figure 2A). A direct correlation between age and self-reported diabetes was observed, and in all the age groups, the prevalence of self-reported diabetes was much higher in the farming population (Supplementary Figure 1). Among the farming community, people directly handling agrochemicals, including pesticide applicators and fertilizer/pesticide mixers, had self-reported diabetes prevalence of 15.2 and 14.2%, respectively, while the self-reported diabetes prevalence among the field workers was 12.2% (Figure 2B). The family history of diabetes among the self-reported diabetes population was 24.6%, and no significant difference (*p* = 0.452) was noticed between the farming and non-farming communities. No significant difference between farming and non-farming populations was observed for sex (*p* = 0.776), daily alcohol intake (*p* = 0.786), smoking (*p* = 0.236), oral tobacco use (*p* = 0.845), and obesity (*p* = 0.521).

On binary logistic regression analysis, among the traditional risk factors for diabetes, only age (*p* < 0.001) and hypertension (*p* < 0.001) showed significant association with high odds ratio for self-reported diabetes in the farming population (Figure 2C). The lesser than expected association of traditional risk factors made us to analyze the association of occupational exposure to agrochemicals with self-reported diabetes prevalence among the farming community. On binary logistic regression analysis, a strong association of exposure to agrochemicals with self-reported diabetes prevalence was observed in all the different exposure categories (pesticide applicators, agrochemical handlers, and field workers) even after adjustment for all confounding variables (Figure 2D). Among the diabetes complications reported in survey, only cardiovascular diseases, kidney failure, cancer, and tuberculosis were validated on cross-reference to medical records in the sub-set of population. A significant association of self-reported diabetes was observed only with CVDs in both farming and non-farming populations (Supplementary Figures 2, 3).

## Discussion

We noticed equal participation of both sexes in the farming activities in the villages of Tamil Nadu, India. Among the age groups, the participation of youth (<35 years) in farming occupation is relatively low, indicating the transition from farming occupation to non-farming occupation among the new generation. Our study shows a nearly two-fold higher prevalence of diabetes in rural farming vs. non-farming communities, seriously raising a research question on the role of agrochemicals in diabetes. As per our previous report (4), there exists around 5–8% of undiagnosed diabetes among rural communities, and hence, the self-reported diabetes survey would have only underestimated the diabetes burden in the rural population. A follow-up study on this rural population will be performed after a period of 3–5 years. A detailed investigation on understanding the molecular mechanism behind the diabetes associated with traditional risk factors like obesity and diabetes associated with EDCs is the need of the hour so that new strategies can be framed to prevent and manage diabetes in the rural world. Among the complications of diabetes, an increased prevalence of kidney failure is associated with farming occupation that is attributed to the increased use of arsenic-rich phosphate fertilizers and glyphosate use. The Sri Lankan Agricultural Nephropathy (SAN) study (11) revealed the association of co-exposure to toxic metals and glyphosate with chronic kidney disease of unknown etiology (CKD-u) among the farming population11. We noticed increased prevalence of cancer among the non-farming communities especially among a few villages in this study, and further investigations revealed the association of coir dyeing and textile industries with cancer incidence in these villages. This study indicates the presence of environmental pollutants of different nature in farming and non-farming villages.

The study has its own limitations. Although the villages for the study were selected based on criteria listed in section “Methods,” the final selection process depended on convenience, access, and contact with the village authorities and willingness of the villagers. The other limitation is that data on dietary habits, physical activity, and education were not collected, but largely, there is no huge variation in these factors within a rural community. The study communities in South India have more intake of carbohydrate diet, which will impact the diabetes prevalence, but this behavior is common to both farming and non-farming communities. In addition, high carbohydrate intake, reduced physical activity due to introduction of machineries in agriculture, non-adherence to diet practices, and medications may also add to the etiology of diabetes in farming community. The self-reported disease status is cross-verified only in 12.8% of the participants, and exposure to agrochemicals is not quantified by measuring the levels of these chemicals in their body fluids.

Altogether, this REDSI study performed among ~0.1 million rural population in Tamil Nadu, South India, indicates the huge burden of diabetes in the rural world. The strong association of occupational exposure to agrochemicals with diabetes in rural communities highlights the need for change in diabetes clinical practice by focusing on occupational safety measures, urging governmental regulatory agencies on safe agricultural policies, and development of new therapeutic strategies targeted at not only understanding the molecular mechanisms but also detoxifying the diabetogenic effect of agrochemicals.

## Data Availability Statement

The original contributions presented in the study are included in the article/Supplementary Material, further inquiries can be directed to the corresponding author/s.

## Ethics Statement

The studies involving human participants were reviewed and approved by the Institutional Ethics Committee of KMCH and Madurai Kamaraj University. The study participants underwent validation provided informed consent to participate in the study.

## Author Contributions

Conceived and designed the experiments: GV, SM, SR, and KS. Acquisition and analysis of data: GV, SM, JC, SKe, BR, SS, AK, SB, AA, PA, TR, MG, JV, VD, SVa, PP, SC, JP, VSS, KA, SVe, GA, SKa, and VG. Statistical analysis of data and interpretation: GV, SM, and KS. Wrote the manuscript: GV. Revised the manuscript: SM, SR, and KS. All authors read and approved the final version of the manuscript.

## Funding

This study was funded by KMCH Research Foundation, India *via* grant no. KMCHRF-RDS, 2017.

## Conflict of Interest

The authors declare that the research was conducted in the absence of any commercial or financial relationships that could be construed as a potential conflict of interest.

## Publisher's Note

All claims expressed in this article are solely those of the authors and do not necessarily represent those of their affiliated organizations, or those of the publisher, the editors and the reviewers. Any product that may be evaluated in this article, or claim that may be made by its manufacturer, is not guaranteed or endorsed by the publisher.
