# Supplementary material for: Association of Agriculture Occupational Exposure With Diabetes and Cardiovascular Risk Factors in South Indian Villages: REDSI Study
Source: Front Cardiovasc Med. 2021 Sep 24;8:737505. doi: 10.3389/fcvm.2021.737505 (PMC8498024; doi:10.3389/fcvm.2021.737505)
Supplement: Supplementary file 1 [file Data_Sheet_1.PDF]

Supplementary Data:

Association of Agriculture Occupational Exposure with Diabetes and Cardiovascular risk factors in South Indian Villages: REDSI Study

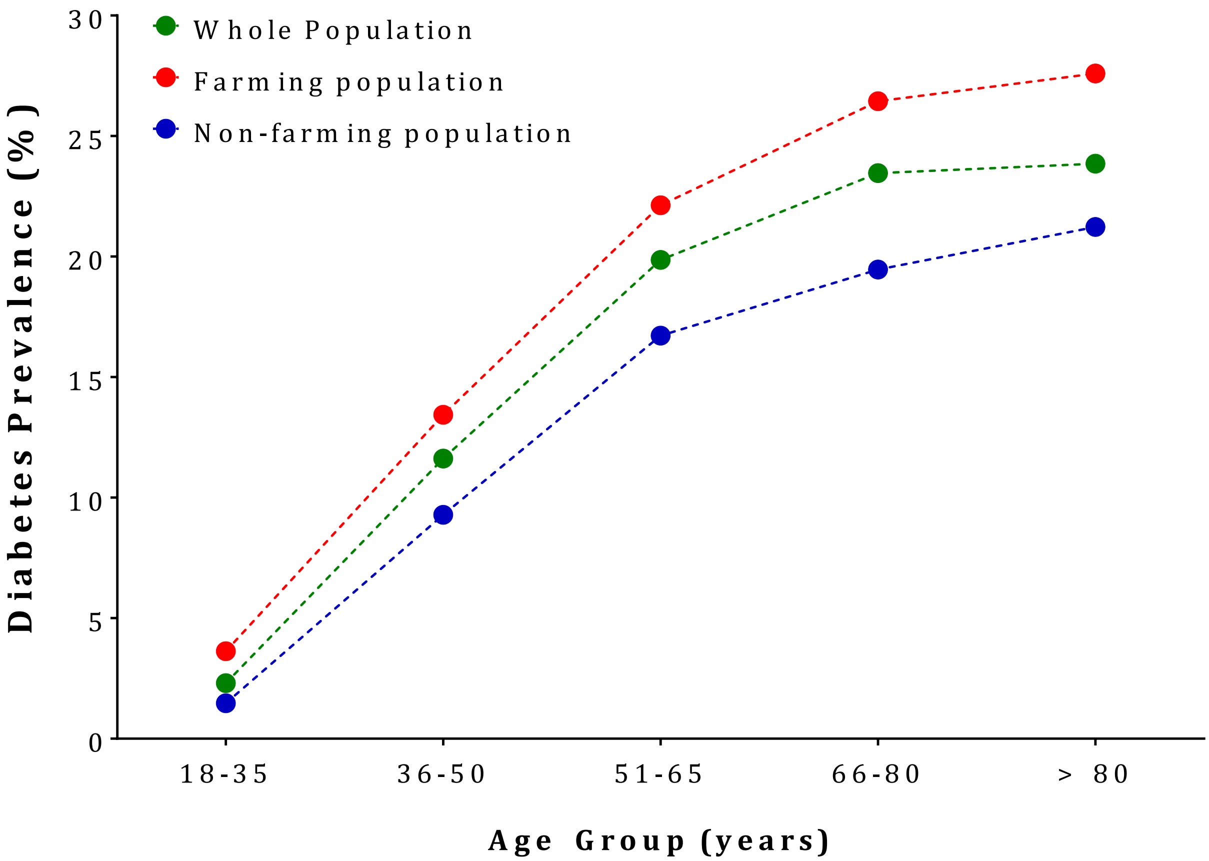

Supplementary Figure S1: Age group-wise prevalence of diabetes among the farming and non-farming rural population

Supplementary Figure S2: Prevalence of diabetic complications among rural population

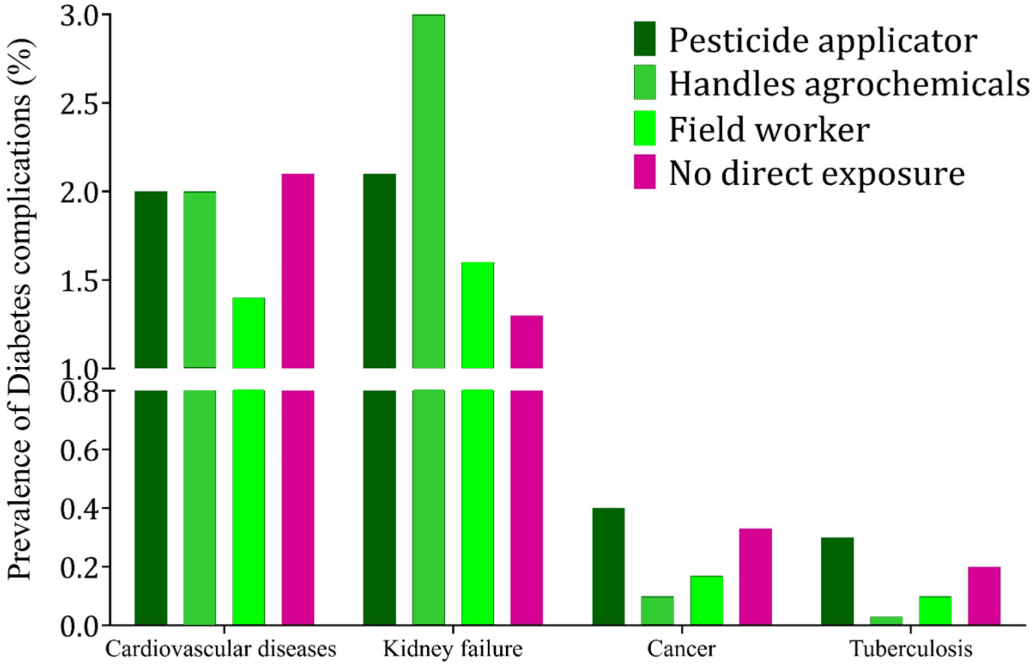

categorized based on their level of exposure to agrochemicals.

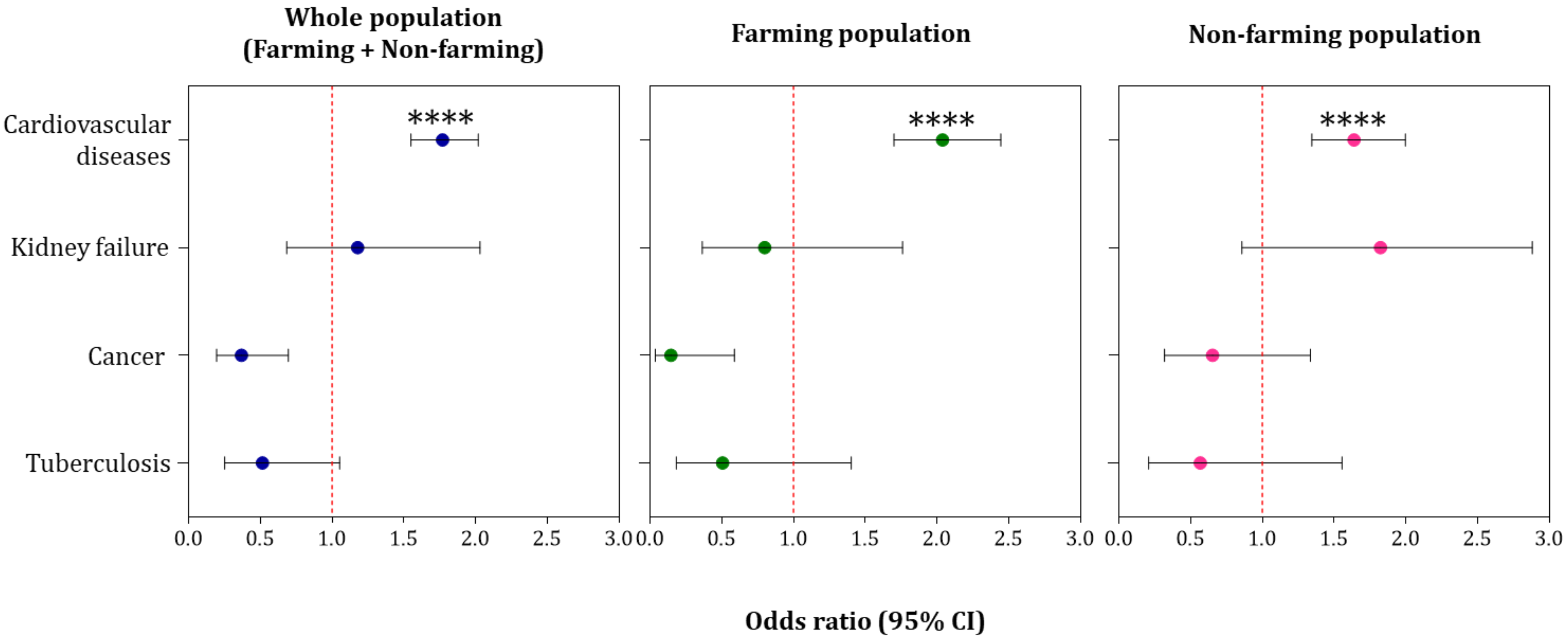

Supplementary Figure S3: Forest plot representing association of diabetes complications with self-reported diabetes in total population, farming and non-farming population. *p<0.05, **p<0.01, ***p<0.001, ****p<0.0001
